# Association Between Diet and Mental Health Outcomes in a Sample of 13,887 Adolescents in Canada

**DOI:** 10.5888/pcd21.240187

**Published:** 2024-10-24

**Authors:** Julia Dabravolskaj, Karen A. Patte, Shelby Yamamoto, Scott T. Leatherdale, Paul J. Veugelers, Katerina Maximova

**Affiliations:** 1 MAP Centre for Urban Health Solutions, Li Ka Shing Knowledge Institute, St. Michael’s Hospital, Toronto, Ontario, Canada; 2 Dalla Lana School of Public Health, University of Toronto, Toronto, Ontario, Canada; 3 Faculty of Applied Health Sciences, Brock University, St. Catharines, Ontario, Canada; 4 School of Public Health, University of Alberta, Edmonton, Alberta, Canada; 5 School of Public Health Sciences, University of Waterloo, Waterloo, Ontario, Canada

## Abstract

**Introduction:**

The high prevalence of mental disorders among adolescents calls for community-based and population-level prevention strategies. Diet is an important intervention target for primary prevention of mental disorders among adolescents. We used data from a large longitudinal study of Canadian adolescents (aged 14–18 y) to examine prospective associations between diet and mental health outcomes.

**Methods:**

We estimated the effect of diet (ie, consumption of vegetables and fruit and sugar-sweetened beverages [SSBs]) at baseline on depressive symptoms, anxiety symptoms, and psychological well-being (measured by the Center for Epidemiologic Studies Depression Scale–Revised, Generalized Anxiety Disorder 7 scale, and Flourishing Scale, respectively) and at 1-year follow-up in a sample of 13,887 Canadian secondary school students who participated in the 2017–2018 and 2018–2019 cycles of the Cannabis, Obesity, Mental health, Physical activity, Alcohol, Smoking, and Sedentary (COMPASS) behavior study. We applied linear mixed-effects methods informed by a directed acyclic graph. Sensitivity analyses assessed the robustness of the effect estimates to unmeasured confounding variables.

**Results:**

Baseline SSB consumption was associated with greater severity of depressive (β = 0.04; 95% CI, 0.01–0.06) and anxiety (β = 0.02; 95% CI, 0–0.05) symptoms, particularly among male students, and poorer psychological well-being (β = −0.03; 95% CI, −0.05 to −0.01) at follow-up. Baseline vegetables and fruit consumption was positively associated with psychological well-being (β = 0.06; 95% CI, 0.03–0.10) but not other mental health outcomes at follow-up.

**Conclusion:**

Our results support the notion that diet should be part of comprehensive mental health prevention and promotion interventions to reduce the prevalence of mental health disorders among adolescents.

**Summary**

**What is already known on this topic?**

Healthy eating might be linked to mental health among adolescents, but the evidence is mostly limited to cross-sectional studies; prospective studies are needed.

**What is added by this report?**

Using longitudinal data, we found that sugar-sweetened beverage (SSB) consumption is prospectively associated with greater severity of depressive and anxiety symptoms and poorer psychological well-being among adolescents, while vegetables and fruit consumption was prospectively associated with better psychological well-being.

**What are the implications for public health practice?**

Community-based and population-level strategies should target SSB and vegetables and fruit consumption to prevent further widening of socioeconomic inequalities in diet, which can translate into widening inequalities in mental health.

## Introduction

In Canada, the mental health burden among adolescents (aged 10–18 y) is high and growing: the prevalence of mood and anxiety disorders increased from 4.3% and 6.0%, respectively, in 2011 to 7.8% and 12.9%, respectively, in 2018 (1), while the rates of mental disorders–related visits to emergency departments rose from 11.7 per 1,000 adolescents in 2003 to 13.5 per 1,000 adolescents in 2009, and then up to 24.1 per 1,000 adolescents in 2017 (2). No effective treatment options currently exist for mental disorders in this age group (3). Considering the far-reaching effects of unmanaged or poorly managed mental disorders later in life (2), there is an urgent need for effective community-based and population-level prevention and promotion strategies to improve mental health among adolescents.

The role of lifestyle behaviors such as regular physical activity, limited screen time (4), and good sleep (5) is well recognized. The evidence supporting the link between diet and mental disorders among adolescents is also accumulating but is less conclusive. Several systematic reviews of observational studies of this age group support the associations of poor dietary intake and eating habits (eg, eating take-away, ultra-processed, and fast foods) with negative mental health outcomes (eg, depression and anxiety) (6–8). The associations reported in cross-sectional studies appeared more consistent than in observation studies; prospective studies are scant.

To support the development of community-based and population-level strategies to improve diet among adolescents, we used data from a large longitudinal study of Canadian adolescents (aged 14–18 y) to examine the association of diet with depressive symptoms, anxiety symptoms, and psychological well-being.

## Methods

### Study design

We used data from the Cannabis, Obesity, Mental health, Physical activity, Alcohol, Smoking, and Sedentary behavior (COMPASS) study (9). The study annually collects survey data from approximately 60,000 students in grades 9 through 12 (age 13–18 y) in secondary schools in British Columbia, Alberta, and Ontario, and Secondary I–V students (aged 12–17 y) in Quebec, Canada. Details on the study are available elsewhere (9). Participants complete the paper-based, anonymous COMPASS questionnaire during class time. For this study, we used data from a subsample of 29,023 students who participated in 2 COMPASS data cycles (2017–2018 and 2018–2019) and whose responses could be longitudinally linked through a deterministic linkage process. Thus, these participants constituted a prospective cohort panel; data gathered in the 2017–2018 cycle were treated as baseline data, and data gathered in the 2018–2019 cycle as 1-year follow-up data. Response proportions for 2017–2018 and 2018–2019 were 81.8% and 84.2%, respectively, with absenteeism on the day of survey administration being the primary reason for nonresponse. We limited analyses to 13,887 participants from 116 schools who had complete data (ie, no missing values) for covariates included in the final models. We observed no major differences between samples of participants included and excluded from analyses.

COMPASS procedures were approved by the University of Waterloo Office of Research Ethics (#30118) and appropriate school board committees, and analyses presented in this study were approved by the Research Ethics Board at the University of Alberta (Pro00119528). Informed consent was obtained from all participants and their legal guardians.

### Measures

Depressive symptoms in the last 7 days were measured by the 10-item Center for Epidemiologic Studies Depression Scale–Revised (CESD-R-10) (10), generalized anxiety disorder symptoms in the last 2 weeks by the 7-item Generalized Anxiety Disorder 7 (GAD-7) scale (11), and psychological well-being by the 8-item Flourishing Scale (12). All scales have been validated among adolescents (10–12). CESD-R-10 scores range from 0 to 30 and GAD-7 from 0 to 21, with higher scores indicating greater severity of symptoms. CESD-R-10 and GAD-7 scores of 10 or higher indicate clinically relevant depressive and anxiety symptoms, respectively. Scores for the Flourishing Scale range from 8 to 40, with higher scores indicating better psychological well-being.

To assess healthy eating, participants reported the number of servings of vegetables and fruit they consumed the day before the survey, including pieces of fresh vegetable or fruit, salad or raw leafy greens, cooked leafy green vegetables, dried or canned or frozen fruit, and 100% fruit or vegetable juice. Participants also reported on their consumption of sugar-sweetened beverages (SSBs) as the number of days during a usual school week and weekend in which they consumed 1) soft drinks (excluding diet or sugar-free drinks), 2) high-energy drinks (eg, Red Bull, Monster, Rockstar), and 3) coffee or tea with sugar (eg, cappuccino, frappuccino, iced tea, iced coffees). Responses for these 3 categories were summed to derive an SSB composite index, ranging from 0 (did not consume any SSBs in the 3 categories) to 21 (consumed SSBs from all 3 categories every day during a usual week) (13).

Participants reported their sex (female, male), age (years), and how much weekly spending money they usually get (CAD$0, $1–$5, $6–$10, $11–$20, $21–$40, $41–$100, >$100). We used responses to additional questions to calculate the following: 1) the average number of minutes of moderate (eg, walking, biking to school, recreational swimming) and vigorous (ie, activities that increase heart rate) physical activity (MVPA) in the last 7 days, 2) hours per day of screen time (ie, watching and/or streaming television shows or movies, playing videos and/or computer games, talking on the telephone, surfing the internet, and texting, messaging, emailing), and 3) hours per day of sleep. For substance use, participants reported the number of days in the last month they smoked 1 or more cigarettes (tobacco smoking) and e-cigarettes (vaping), and the past 12-month frequency of having 5 or more alcoholic drinks on 1 occasion (binge drinking) and using cannabis. Current users of cigarettes and e-cigarettes were those reporting smoking or vaping at least once in the past 30 days, and current cannabis users and binge drinkers were those reporting these behaviors at least once per month in the past year. Eating behaviors included skipping breakfast (ie, a negative response to “I eat breakfast every day”) and weight-loss attempts (ie, a positive response to “I’m trying to lose weight”) as a proxy for dieting behaviors. To assess social support, participants were asked to indicate their level of agreement (strongly agree, agree, neither agree nor disagree, disagree, strongly disagree) with 2 statements: “My social relationships are supportive and rewarding” (supportive social relationships) and “I have a happy home life” (happy family life). Responses of “strongly agree” were combined with “agree,” and responses of “neither agree nor disagree” were combined with “disagree” and “strongly disagree.”

Additionally, we used the following 5 variables in sensitivity analyses: self-concept, self-perceived weight status, race and ethnicity, academic achievement, and geographic area of residence. Self-concept was measured by the Self-Description Questionnaire II (14): participants were asked to rate the following statements on a 5-point Likert scale (1 corresponding to true and 5 to false): “in general, I like the way I am”; “overall, I have a lot to be proud of”; “a lot of things about me are good”; “when I do something, I do it well”; and “I like the way I look.” Responses were summed to derive continuous scores ranging from 0 to 25, with higher scores indicating poorer self-concept. For self-perceived weight status, participants responded to the question “How do you describe your weight?” with the following options: very or slightly underweight, about right, and slightly or very overweight. We combined very and slightly underweight into the underweight category, and very and slightly overweight into the overweight category. Race and ethnic categories were Asian, Black, Hispanic, White, and other. For academic achievement, the survey asked about an overall mark in a current or recent math course (90%–100%, 80%–89%, 70%–79%, 60%–69%, 55%–59%, 50%–54%, and <50%). Categories for geographic area were census metropolitan areas (>100,000 population), census agglomerations (10,000 to 100,000 population), and small towns and rural areas (<10,000 population).

### Statistical analyses

We used mean (SD) for normally distributed variables and median and median absolute deviation (MAD) otherwise to summarize data for continuous variables (ie, age; number of servings of vegetables and fruit and SSBs; scores on the CESD-R-10, the GAD-7, the Flourishing Scale, and the Self-Description Questionnaire II; minutes per day of MVPA; and hours per day of screen time and sleep). Responses with 3 SDs or more outside the sample mean or in excess of 24 hours (for combined screen time and sleep) were considered outliers and winsorized (a method of averaging that replaces the smallest and largest observations with the values closest to them). Although the intraclass correlation coefficients for all outcomes were <0.5, we chose mixed-effects models to account for the hierarchical data structure. We applied complete case analysis and fitted linear mixed-effects models with a random school intercept using maximum likelihood estimation to estimate the effects of vegetables and fruit and SSB consumption at baseline on depressive and anxiety symptoms and psychological well-being at 1-year follow-up. To inform the choice of confounders relevant to the diet–mental health relationship for which we adjusted, we developed a causal model (directed acyclic graph) for this focal relationship. Because we considered mental health at baseline as an important confounder, we followed a suggestion by Tennant et al (15) and conducted the follow-up adjusted for baseline analysis rather than modeling the change between mental health at follow-up and baseline.

All covariates were measured at baseline. The final models were adjusted for mental health at baseline, weekly spending money, age, sex, breakfast skipping, weight loss attempts, physical activity, screen time, sleep, tobacco (smoking cigarettes and vaping), binge-drinking, cannabis use, supportive social relationships, and happy family life. The final models were stratified by sex, given existing sex differences in diet and mental health. We used Stata version 17 (StataCorp LLC) to perform all analyses.

### Sensitivity analyses

Sensitivity analyses for unmeasured confounding included estimating E-values for regression coefficients reported in the final models, where 95% CIs did not include the null. For this analysis, we used the web-based E-value calculator (16). We considered self-concept as a positive exposure control because it is causally linked to mental health problems in adolescence (17), and the association between self-concept and mental health might be similarly confounded (eg, by lifestyle behaviors, social support, baseline mental health). Lastly, to examine whether the addition of other covariates impacts effect estimates, we added self-perceived weight status, race and ethnicity, academic achievement, and geographic area to the final models.

## Results

The mean (SD) age of participants was 14.9 (1.2) years at baseline and 15.8 (1.2) years at follow-up. At baseline, most participants were White (77.9%), and two-thirds (67.0%) lived in census metropolitan areas (Table 1).

**Table 1:** Characteristics at Baseline of 13,887 Adolescents Participating in the COMPASS Study, Canada, 2017–2018^a^

Characteristic	Total	Female	Male
**Variables used in primary analysis**			
Depressive symptoms, measured by CESD-R-10^b^			
Score, median (MAD)	7.0 (4.4)	8.0 (5.9)	6.0 (4.4)
Score of ≥10, n (%)	4,504 (32.4)	3,028 (40.7)	1,476 (22.9)
Anxiety symptoms, measured by GAD-7^c^			
Score, median (MAD)	5.0 (4.4)	6.0 (5.9)	3.0 (4.4)
Score of ≥10, n (%)	3,157 (22.7)	2,311 (31.0)	846 (13.1)
Psychological well-being, measured by the Flourishing Scale^d^			
Score, median (MAD)	32.0 (5.9)	32.0 (5.9)	33.0 (4.5)
Vegetables and fruit, median (MAD), servings per day	3.0 (1.5)	3.0 (1.5)	3.0 (1.5)
SSB composite index,^e^ median (MAD)	3.0 (2.9)	3.0 (2.9)	3.0 (2.9)
0	2,601 (18.7)	1,520 (20.4)	1,081 (16.8)
>0	11,286 (81.3)	5,928 (79.6)	5,358 (83.2)
Sex, n (%)	—	7,448 (54)	6,439 (46)
Age, mean (SD)	14.9 (1.2)	14.9 (1.2)	14.9 (1.1)
Weekly spending money in Canadian dollars, n (%)			
0	3,049 (22.0)	1,473 (19.8)	1,576 (24.5)
1**–**20	4,851 (34.9)	2,709 (36.3)	2,142 (33.3)
21**–**100	3,775 (27.2)	2,132 (28.7)	1,643 (25.5)
**>**100	2,212 (15.9)	1,134 (15.2)	1,078 (16.7)
Weight-loss attempt, n (%)	609 (4.4)	476 (6.4)	133 (2.1)
Breakfast skipping, n (%)	7,287 (52.5)	3,522 (47.3)	3,765 (58.5)
MVPA, median (MAD), minutes per week	90.0 (66.7)	83.6 (60.4)	100.7 (73.1)
Screen time, median (MAD), hours per day	6.0 (3.3)	5.8 (3.3)	6.0 (3.3)
Sleep, median (MAD), hours per day	7.8 (1.1)	7.5 (1.1)	8.0 (1.5)
Current use of cigarettes, n (%)	672 (4.8)	330 (4.4)	342 (5.3)
Current use of e-cigarettes, n (%)	2,364 (17.0)	1,104 (14.8)	1,260 (19.6)
Current binge drinking, n (%)	1,611 (11.6)	827 (11.1)	784 (12.2)
Current cannabis use, n (%)	1,016 (7.3)	504 (6.7)	512 (7.9)
Supportive social relationships,^f^ n (%)			
Strongly agree or agree	10,853 (78.1)	5,721 (76.8)	5,132 (79.7)
Neither agree nor disagree, disagree, or strongly disagree	3,034 (21.9)	1,727 (23.2)	1,307 (20.3)
Happy home life,^g^ n (%)			
Strongly agree or agree	11,173 (80.5)	5,711 (76.7)	5,562 (86.4)
Neither agree nor disagree, disagree, or strongly disagree	2,714 (19.5)	1,737 (23.3)	887 (13.6)
**Variables used in sensitivity analyses**			
Self-concept score,^h^ mean (SD)	10.0 (4.4)	11.0 (4.4)	9.0 (4.4)
Self-perceived weight status, n (%)			
Underweight	2,222 (16.0)	821 (11.0)	1,401 (21.8)
About right	8,427 (60.7)	4,714 (63.3)	3,713 (57.7)
Overweight	3,238 (23.3)	1,913 (25.7)	1,325 (20.6)
Race and ethnicity, n (%)^i^			
White	10,818 (77.9)	5,837 (78.4)	4,981 (77.4)
Black	526 (3.8)	261 (3.5)	265 (4.1)
Asian	1,911 (13.8)	1,040 (14.0)	871 (13.5)
Hispanic	467 (3.4)	250 (3.4)	217 (3.4)
Other	1,406 (10.1)	805 (10.8)	601 (9.3)
Overall grade in a current or recent math course, n (%)			
90%–100%	3,664 (26.4)	2,097 (28.2)	1,567 (24.3)
80%–89%	4,072 (29.3)	2,222 (29.8)	1,850 (28.7)
70%–79%	3,077 (22.2)	1,584 (21.3)	1,493 (23.2)
60%–69%	1,739 (12.5)	883 (11.9)	856 (13.3)
<60%	1,335 (9.6)	662 (8.8)	673 (10.5)
Geographic area^j^, n (%)			
Census metropolitan areas	9,306 (67.0)	5,042 (67.7)	4,264 (66.2)
Census agglomerations	1,914 (13.8)	1,008 (13.5)	906 (14.1)
Small towns and rural areas	2,667 (19.2)	1,398 (18.8)	1,269 (19.7)

Abbreviations: CESD-R-10, Center for Epidemiologic Studies Depression Scale Revised-10; COMPASS, Cannabis, Obesity, Mental health, Physical activity, Alcohol, Smoking, and Sedentary behavior; GAD-7, Generalized Anxiety Disorder-7; MAD, median absolute deviation; MVPA, moderate-to-vigorous physical activity; SSB, sugar-sweetened beverage.

a Data were from the COMPASS study, which annually collects survey data from approximately 60,000 students in grades 9 through 12 (aged 13–18 y) in secondary schools in British Columbia, Alberta, and Ontario, and Secondary I–V students (aged 12–17 y) in Quebec, Canada (9).

b CESD-R-10 scores range from 0 to 30 with higher scores indicating greater severity of symptoms; scores ≥10 indicate clinically relevant depressive symptoms (10).

c GAD-7 scores range from 0 to 21, with higher scores indicating greater severity of symptoms; scores ≥10 indicate clinically relevant anxiety symptoms (11).

d Scores for the Flourishing Scale range from 8 to 40, with higher scores indicating better psychological well-being (12).

e Responses for these 3 categories of SSBs were summed to derive a SSB composite index, ranging from 0 (did not consume any SSBs in the 3 categories) to 21 (consumed SSBs from all 3 categories every day during a usual week) (13).

f Participants were asked to indicate their level of agreement with the following statement: “My social relationships are supportive and rewarding.” Response options were strongly agree, agree, neither agree nor disagree, disagree, and strongly disagree.

g Participants were asked to indicate their level of agreement with following statement: “I have a happy home life.” Response options were strongly agree, agree, neither agree nor disagree, disagree, and strongly disagree.

h Measured by the Self-Description Questionnaire II (14).

i Proportions do not add to 100 because participants could choose multiple races and ethnicities.

j Geographic area was classified according to population into census metropolitan areas (>100,000 population), census agglomerations (10,000 to 100,000 population), and small towns and rural areas (<10,000 population).

At baseline, more than one-third of participants (43.1%) reported having $21 or more in weekly spending money. For the CESD-R-10 and GAD-7 scales, 32.4% and 22.7% of participants, respectively, were classified as having clinically significant depressive and anxiety symptoms. Both depressive and anxiety symptoms were more common among female students than male students (40.7% vs 22.9% for depressive symptoms and 31.0% vs 13.1% for anxiety symptoms). We found no sex-based differences in psychological well-being (median score for Flourishing Scale = 32.0), number of servings per day of vegetables and fruit (median = 3.0), or SSB composite index (median = 3.0). Male students reported being physically active, on average, 17 minutes per day more than female students (100.7 vs 83.6 minutes per day of MVPA) and using screens, on average, approximately 15 minutes per day more than female students (6.0 vs 5.8 hours per day). Substance use was more common among male students: 5.3% of male versus 4.4% of female students were classified as current smokers, 19.6% versus 14.8% as current users of e-cigarettes, 12.2% versus 11.1% as current binge-drinkers, and 7.9% versus 6.7% as current users of cannabis. More female than male students reported weight-loss attempts (6.4% vs 2.1%), and more male than female students reported skipping breakfast (58.5% vs 47.3%). Male students reported greater agreement than female students with the statements that their social relationships were supportive (79.7% vs 76.8% agreed or strongly agreed) and home life was happy (86.4% vs 76.7% agreed or strongly agreed).

### Mixed-effects modeling

We observed no prospective association of vegetables and fruit consumption with the severity of depressive (β = 0.03; 95% CI, −0.02 to 0.07) or anxiety symptoms (β = 0.03; 95% CI, −0.01 to 0.07), whereas it was positively associated with psychological well-being (β = 0.06; 95% CI, 0.03–0.10) at follow-up (Table 2). SSB consumption was associated with greater severity of depressive (β = 0.04; 95% CI, 0.01–0.06) and anxiety symptoms (β = 0.02; 95% CI, 0–0.05), particularly among male students, and poorer psychological well-being (β = −0.03; 95% CI, −0.05 to −0.01) at follow-up. Adjustment for mental health at baseline yielded the biggest reduction in Akaike information criteria values and effect estimates, with adjustment for other confounders moving effect estimates closer to the null.

**Table 2:** Associations Between Dietary Variables and Self-Concept at Baseline (2017–2018) and Mental Health at 1-Year Follow-Up (2018–2019) Among 13,887 Adolescents Participating in the COMPASS Study, Canada^a^

Variable	Unadjusted model	Adjusted model^b^	Sensitivity analyses	
			Model 1^c^	Model 2^d^
**Depressive symptoms**				
Exposure of interest				
Consumption of vegetables and fruit^e^	−0.24 (−0.29 to −0.19)^f^	0.03 (−0.02 to 0.07)	0.03 (−0.01 to 0.07)	0.03 (−0.01 to 0.07)
SSB composite index^g^	0.17 (0.14 to 0.20)^f^	0.04 (0.01 to 0.06)^f^	0.04 (0.01 to 0.06)^f^	0.04 (0.01 to 0.06)^f^
Positive exposure control				
Self-concept^h^	0.68 (0.66 to 0.70)^f^	0.19 (0.17 to 0.22)^f^	0.19 (0.16 to 0.21)^f^	0.19 (0.16 to 0.21)^f^
**Anxiety symptoms**				
Exposure of interest				
Consumption of vegetables and fruit^e^	−0.12 (−0.17 to −0.07)^f^	0.03 (−0.01 to 0.07)	0.03 (0 to 0.07)	0.04 (0 to 0.07)
SSB composite index^g^	0.15 (0.12 to 0.17)^f^	0.02 (0 to 0.05)^f^	0.02 (0 to 0.05)^f^	0.02 (0 to 0.05)^f^
Positive exposure control				
Self-concept^h^	0.53 (0.52 to 0.55)^f^	0.12 (0.10 to 0.15)^f^	0.12 (0.10 to 0.14)^f^	0.12 (0.10 to 0.14)^f^
**Psychological well-being**				
Exposure of interest				
Consumption of vegetables and fruit^e^	0.45 (0.41 to 0.50)^f^	0.06 (0.03 to 0.10)^f^	0.06 (0.02 to 0.10)^f^	0.06 (0.02 to 0.10)^f^
SSB composite index^g^	−0.11 (−0.14 to −0.08)^f^	−0.03 (−0.05 to −0.01)^f^	−0.03 (−0.05 to −0.01)^f^	−0.03 (−0.05 to −0.01)^f^
Positive exposure control				
Self-concept^h^	−0.70 (−0.71 to −0.68)^f^	−0.21 (−0.23 to −0.18)^f^	−0.21 (−0.23 to −0.18)^f^	−0.20 (−0.23 to −0.18)^f^

Abbreviations: COMPASS, Cannabis, Obesity, Mental health, Physical activity, Alcohol, Smoking, and Sedentary behavior; SSB, sugar-sweetened beverage.

a Data were from the COMPASS study, which annually collects survey data from approximately 60,000 students in grades 9 through 12 (aged 13–18 y) in secondary schools in British Columbia, Alberta, and Ontario, and Secondary I–V students (aged 12–17 y) in Quebec, Canada (9). All values are β (95% CI); βs are the unstandardized β-coefficients from the linear mixed-effects models.

b Adjusted models were adjusted for depressive and anxiety symptoms or psychological well-being at baseline (as appropriate), weekly spending money, age, sex, breakfast skipping, weight loss attempts, physical activity, screen time, sleep, smoking cigarettes and e-cigarettes, binge-drinking, cannabis use, supportive social relationships, and happy family life.

c Additionally adjusted for self-perceived weight status.

d Additionally adjusted for race and ethnicity, geographic location, and academic achievement.

e Participants reported the number of servings of vegetables and fruit they consumed the day before the survey.

f Estimates in which 95% CIs do not include 0.

g Responses for these 3 categories of SSBs were summed to derive a composite SSB index, ranging from 0 (did not consume any SSBs in the 3 categories) to 21 (consumed SSBs from all 3 categories every day during a usual week) (13).

h Self-concept was measured by the Self-Description Questionnaire II (14). Participants were asked to rate 5 statements on a 5-point Likert scale (1 corresponding to true and 5 to false): “in general, I like the way I am”; “overall, I have a lot to be proud of”; “a lot of things about me are good”; “when I do something, I do it well”; and “I like the way I look.” Responses were summed; score ranges from 0 to 25, with higher scores indicating poorer self-concept.

### Sensitivity analyses

All E-values for point estimates were higher than 1.06 on the risk ratio scale (Table 3), suggesting that unmeasured confounder(s) would have to be relatively weak to nullify the associations. For example, an unmeasured confounder should be associated with both vegetables and fruit consumption and psychological well-being by a risk ratio of at least 1.11-fold to nullify the association and by at least 1.06-fold to shift the 95% CI to include the null. Also, the associations between self-concept and mental health outcomes remained strong, and effect estimates were considerably larger than those obtained for the exposures of interest. Finally, the addition of other covariates (ie, self-perceived weight status, race and ethnicity, academic achievement, and geographic area) did not affect the estimates for any of the associations (Table 2).

**Table 3:** Sensitivity Analyses Examining the Robustness of Effect Estimates Reported in Table 2 to Unmeasured Confounding in a Sample of 13,887 Adolescents Participating in the COMPASS Study, Canada

Variable	Adjusted model, β (SE) [95% CI]	E-value^a^ for point estimate	E value^a^ for 95% CI
**Depressive symptoms**			
SSB composite index^b^	0.04 (0.01) [0.01 to 0.06]	1.08	1.06
**Anxiety symptoms**			
SSB composite index^b^	0.02 (0.01) [0 to 0.05]	1.06	1.01
**Psychological well-being**			
Vegetables and fruit	0.06 (0.02) [0.03 to 0.10]	1.11	1.06
SSB composite index^b^	−0.03 (0.01) [−0.05 to −0.01]	1.08	1.04

Abbreviations: CESD-R-10, Center for Epidemiologic Studies Depression Scale Revised-10; COMPASS, Cannabis, Obesity, Mental health, Physical activity, Alcohol, Smoking, and Sedentary behavior; GAD-7, Generalized Anxiety Disorder-7; SSB, sugar-sweetened beverage.

a To calculate E-values, the following SDs for the outcomes of interest were used: 6.01 for the CESD-R-10 score, 5.55 for the GAD-7 score, and 5.49 for the Flourishing Scale score at 1-year follow-up. E-values are reported on the risk ratio scale.

b Responses for these 3 categories of SSBs were summed to derive a composite SSB index, ranging from 0 (did not consume any SSBs in the 3 categories) to 21 (consumed SSBs from all 3 categories every day during a usual week) (13).

## Discussion

In this longitudinal study of 13,887 Canadian adolescents, we found that greater SSB consumption was associated with greater severity of depressive and anxiety symptoms and poorer psychological well-being. The findings for vegetables and fruit consumption were less consistent: more consumption was not associated with the severity of depressive or anxiety symptoms but was associated with better psychological well-being. We found no substantial sex differences, in line with other prospective cohort studies (18,19). Our sensitivity analyses highlight the importance of adjusting for mental health at baseline when analyzing the associations between diet and mental health.

Existing literature on the association between SSB consumption and mental health outcomes among adolescents is dominated by cross-sectional studies. For example, a cross-sectional study in a sample of 1,311 children and adolescents showed that SSB consumption of 1 or more servings per day was associated with greater severity of depressive symptoms (odds ratio [OR] = 2.28; 95% CI, 1.30–4.01) (20). Another cross-sectional study in a much larger sample of 13,486 children and adolescents reported that those who consumed SSBs daily had 1.41 (95% CI, 1.23–1.61) and 1.47 (95% CI, 1.29–1.69) times higher odds of reporting depressive and anxiety symptoms, respectively (21). To our knowledge, our study is unique in that it reports on the *prospective* associations between SSB consumption and mental health outcomes among Canadian adolescents. Our findings offer an exciting avenue for mental health promotion among adolescents as they gain independence and begin to make decisions about their diet and eating behaviors that have long-term effects on their physical and mental health (22).

Although the magnitude of the associations between SSB consumption and all mental health outcomes weakened after adjusting for all confounders, with a particularly notable change in estimates after the adjustment for mental health at baseline (consistent with the literature [19]), all associations remained significant, albeit small. However, small effect estimates do not mean that there is evidence of no effect (23). In fact, we argue that small effect estimates are to be expected for at least 2 reasons. First, mental health is influenced by a multitude of intertwined factors that act at multiple levels (individual, family, social). Second, it might take time for the effect of diet on mental health to accumulate and be observed. Hence, examining this relationship through the life-course epidemiology lens merits attention.

Finally, diet often co-occurs with other behavioral factors that are causally related to mental health (eg, tobacco smoking, binge drinking [24], vaping [25], cannabis use [26], lack of physical activity, excess screen time [27], and poor sleep [5]), and the combined effect of these lifestyle behaviors on mental health might compound in a synergistic fashion. This co-occurrence of behaviors underscores the importance of developing and implementing comprehensive mental health prevention interventions that target multiple behaviors in adolescence, when common mental disorders often manifest for the first time (28) and when long-term health-related behaviors are being established (29). Moreover, our findings should be viewed in the context of an alarmingly high prevalence of mental health problems and unhealthy lifestyle behaviors reported in this large population-based study of secondary school students. According to established cutoffs, more than 30% and 20% of participants reported having clinically significant depressive and anxiety symptoms, respectively, with both being almost twice as common among female students than male students. Moreover, half of the participants reported consuming fewer than 3 servings of vegetables and fruit the day before the survey, 4 in 5 reported consuming at least 1 SSB drink on at least 1 day per week, 1 in 5 reported vaping in the past month, and almost 1 in 10 reported binge drinking and cannabis use in the past year.

Our results suggest that comprehensive community-based and population-level mental health promotion strategies for adolescents, and specifically strategies focused on diet, can be improved by reinforcing the importance of creating health-supporting environments to enable behavior change. In Canada, an estimated 17.5% of free sugar originates from SSBs (30), and given the enormous economic burden of free sugar consumption (31), improving existing policy interventions, such as sugar taxation and other priority interventions (eg, product labeling, subsidies for healthful foods) (32), should be advocated for. We recently demonstrated that the COVID-19 pandemic exacerbated income inequalities in lifestyle behaviors, including dietary intake (33). If not addressed, these inequalities in lifestyle behaviors will translate into widening inequalities in mental health in the years to come.

### Limitations

Our study has several potential limitations. First, while drawing causal inferences from observational data is challenging, randomized controlled trials are neither ethical nor feasible for studying the effect of diet on mental health, making high-quality prospective cohort studies the only avenue for causal inference in this field. Next, we attempted to create a comprehensive causal model to inform the selection of confounders, but we encourage researchers to develop other causal models and consider other potential confounders. For example, Molendijk et al (34) previously highlighted metabolic diseases as a potentially important confounder to consider because they may play a role in the etiology of mental disorders. Additionally, important covariates at the parental level (eg, parental mental health, eating habits, health literacy) may exist, but they would likely affect both diet and mental health through socioeconomic position, and the latter, socioeconomic position, was adjusted for in our study.

Addressing measurement error, particularly associated with dietary intake, is a difficult task. Comprehensive dietary measures would help reduce the measurement error and allow us to consider other approaches to conceptualizing exposures (eg, diet quality, dietary patterns). Unfortunately, we did not locate any studies in Canada that collect comprehensive dietary data, particularly in combination with mental health data, from adolescents. Thus, we relied on self-reported consumption of vegetables and fruit the day before the survey and the number of days during a usual week on which SSBs were consumed as our exposures of interest. Moreover, weekly spending money, weight-loss attempts, and the presence of supportive social relationships and a happy family life are imperfect indicators of socioeconomic position, dieting behaviors, and social support, respectively. Additionally, all measures were self-reported and therefore recall bias is possible, although it might have been partially negated by the anonymous nature of the COMPASS questionnaire. Given the large sample size, analyses could be overpowered; therefore, *P* values did not guide interpretation of the results.

### Conclusion

Our results from a large longitudinal study with unique measures on a full range of lifestyle behaviors and mental health among adolescents showed that SSB consumption is consistently associated with greater severity of depressive and anxiety symptoms, as well as poorer psychological well-being, while greater consumption of vegetables and fruit is associated with better psychological well-being. These findings support the need for targeting diet, and specifically consumption of SSBs and vegetables and fruit, as part of comprehensive strategies for improving community-based and population-level mental health.
